# Early Diagnosis of Abnormal Left Ventricular Systolic Functions of Rare Pathogenic Titin Mutation Gene Carriers in FHCM by Three-Dimensional Speckle Tracking Echocardiography Combined with Gene Detection

**DOI:** 10.1155/2022/3415545

**Published:** 2022-10-11

**Authors:** Xiang-hong Luo, Rui Zhu, Qian Chen, Pei-hong Shi, Li-sha Na

**Affiliations:** ^1^Department of Cardiology, Wuzhong People's Hospital, Wuzhong City, Ningxia 751100, China; ^2^Department of Cardiac Function Examination of Heart Centre, General Hospital of Ningxia Medical University, Yinchuan City, Ningxia 750004, China; ^3^Echocardiography and Vascular Ultrasound Center, The First Affiliated Hospital, Zhejiang University School of Medicine, Hangzhou City, Zhejiang 310003, China; ^4^Department of Ultrasonic Imaging, Wuzhong People's Hospital, Wuzhong City, Ningxia 751100, China

## Abstract

**Objective:**

This study aimed to explore the early diagnosis of abnormal left ventricular systolic function of rare pathogenic titin (TTN) mutation gene carriers in familial hypertrophic cardiomyopathy (FHCM) by three-dimensional speckle tracking echocardiography (3D-STE) combined with gene detection.

**Methods:**

Eighteen members of a Hui nationality family in Ningxia province of China were enrolled in this study in July 2019. The proband was tested with high-throughput sequencing of gene detection technology to detect the whole exome, and the mutation locus of pathogenic TTN gene was analyzed. According to the result, 16 subjects were divided into two groups: carrier group (*n* = 4) and noncarrier group (*n* = 12). Related indicators from 2DE were obtained, and myocardial strain indicators from 3D-STE were analyzed by postprocessing software of Tomtec. Strain indicators included global longitudinal strain (GLS), global circumference strain (GCS), global radial strain (GRS), regional longitudinal strain (RLS), regional circumference strain (RCS), and regional radial strain (RRS). All those indicators were compared between the two groups, and a receiver operating characteristic (ROC) curve was used for further analysis.

**Results:**

There were 4 subjects diagnosed as asymptomatic TTN gene carriers with the mutation locus of Val135643Ile. Compared with the noncarrier group, GLS and partial RLS were significantly reduced in the carrier group. The ROC curve shows that GLS has the largest AUC, and its sensitivity was better than LVPWD and specificity was better than IVSD and LVMI obtained from 2DE in the carrier group.

**Conclusions:**

There were 4 subjects diagnosed as asymptomatic TTN gene carriers with the mutation locus of Val135643Ile, and their GLS and partial RLS were significantly reduced; GLS had the better sensitivity and specificity than LVPWD, IVSD, and LVMI.

## 1. Introduction

Hypertrophic cardiomyopathy (HCM) is a kind of heart disease caused mainly by dominant mutations in the sarcomere gene [1, 2], and approximately 50% of the offspring of people with HCM carrying the pathogenic gene are likely to develop the disease. The pathophysiological changes and clinical manifestations of HCM are diverse, with age-dependent penetrance. In the early stages, patients usually have normal ventricular wall thickness [3, 4], which gradually increases as the disease progresses and can cause severe arrhythmias, sudden cardiac death, and refractory heart failure in the later stages [5]. The three-dimensional speckle tracking echocardiography (3D-STE) technique can sensitively identify early myocardial systolic abnormalities in patients with HCM, which, together with gene detection, can not only greatly improve the diagnostic efficiency but also allows for the identification of asymptomatic pathogenic gene carriers at the subclinical stage. In the present study, 18 members of a Hui family, from Ningxia province, with familial HCM (FHCM) were investigated with the application of 3D-STE combined with gene detection. Firstly, asymptomatic disease gene carriers and noncarriers were screened by gene detection technology. Then, we determined whether asymptomatic disease gene carriers in the family have abnormal left ventricular systolic function. Therefore, the relevant parameters of 2DE and 3D-STE left ventricular global and segmental systolic strain were compared, and the relevant parameters of abnormalities were identified, analyzed, and discussed. The present study aims to provide an objective and accurate ultrasonic diagnostic basis for early diagnosis, screening, and regular follow-up of FHCM patients.

## 2. Materials and Methods

### 2.1. Study Subjects

**Figure 1:**
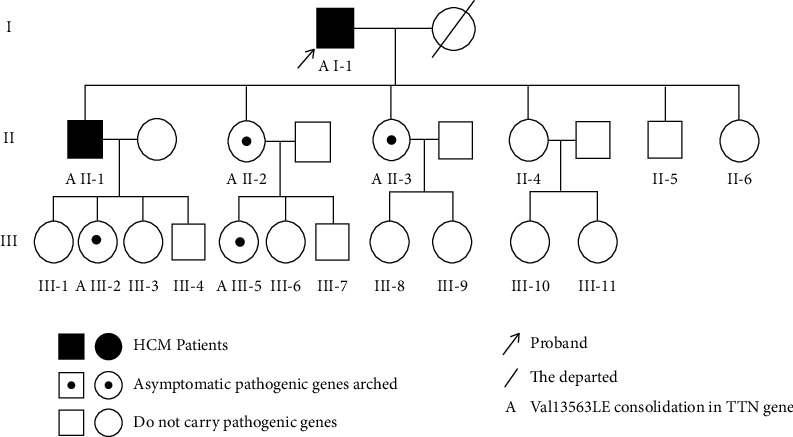
Genogram of the carrier with mutations in the TTN pathogenic gene.

A Hui pedigree from Ningxia province, including the proband and their immediate relatives, totaling 18 individuals across 3 generations, who visited the General Hospital of Ningxia Medical University in July 2019 (Figure 1), were selected as the study subjects. All subjects were ≥18 years of age. The diagnostic criteria for HCM are as follows: according to the recommendations in the 2014 Guidelines for the Diagnosis and Treatment of HCM by the European Society of Cardiology [6], HCM in adults is diagnosed with a finding of a thickness ≥15 mm in one or more segments of the left ventricular wall detected by any imaging method (including routine two-dimensional echocardiogram, cardiac magnetic resonance imaging, and computed tomography), which cannot be explained by overload alone. For the first-degree relatives of patients with HCM, in the absence of other known causes, the diagnosis is confirmed by cardiac imaging showing a thickness of ≥13 mm in one or more segments of the left ventricular wall. In the present study, after gene detection and echocardiographic examination, two patients were confirmed with HCM with a left ventricular ejection fraction (LVEF) that was >50%, without any obstruction of the left ventricular outflow. Four subjects were asymptomatic pathogenic gene carriers with an LVEF that was >50%.

The remaining 12 subjects had negative results in the gene detection, normal electrocardiogram, and echocardiographic indicators and no cardiovascular-related clinical manifestations. Through medical and family histories investigation, genetic testing, and relevant examinations, the cases of left ventricular myocardial hypertrophy and myocardial damage caused by other known reasons in all the family members were excluded, including ① systemic or other systemic diseases that might cause cardiac hypertrophy; ② other heart diseases (e.g., coronary artery disease, pulmonary artery disease, congenital heart disease, valvular heart disease, or myocardial densification insufficiency) and cardiac arrhythmias; ③ previous surgeries related to ablation or resection of the ventricular septum and ICD implantation; ④ currently receiving treatment related to cardiovascular diseases or related treatment affecting cardiac function, such as radiotherapy and chemotherapy; and ⑤ being an athlete with cardiac hypertrophy. All the subjects signed informed consent. The members of the family were divided into two groups according to whether they carried the giant skeletal muscle protein titin (TTN) Val135643Ile gene mutation or not, resulting in a group with four cases carrying the pathogenic gene (the carrier group) and a group with 12 subjects not carrying the pathogenic gene (the noncarrier group).

### 2.2. Gene Detection Analysis

10 ml of the peripheral venous blood was drawn from the proband with HCM, and the genomic DNA was extracted after anticoagulation. A high-performance ultrasonic sample processing system was used, and the DNA samples were randomly broken, sorted, and screened to prepare a hybridization library. Capture enrichment was conducted with Xeon chips.

High-throughput sequencing of each qualified library was performed on the BGISEQ-500 platform. Using Burrows–Wheeler Aligner (BWA V0.7.15) alignment software, each sample was aligned to the human reference genome sequence (GRCh37/HG19) to target the pathogenic mutation loci of the proband. Subsequently, all other members of the family were validated for specific mutations using the Sanger method. Genomic DNA was extracted in the same way as described above, and primers were designed together with the application of the polymerase chain reaction resequencing and amplification techniques to verify the presence of mutated loci and identify the presence of identical mutated loci, thereby identifying the carriers of the HCM pathogenic gene in the tested subjects (see Figure 1).

### 2.3. Apparatus and Methods

#### 2.3.1. Apparatus

The following apparatus was used: the Doppler ultrasound diagnostic system (Philips iE33 Doppler ultrasound diagnostic system: name, iE33, manufacturer: Philips Ultrasound Co. Ltd.; production area: USA; launched: 2017; S5-1/X5-1: ultrasonic probe launched simultaneously with iE33), an S5-1 phased array probe (1–5 MHz), an X5-1 matrix volume probe (1–5 MHz), and 3D ultrasound image processing software (Tomtec software: name, Tomtec; manufacturer: Philips Ultrasound Co. Ltd.; production area: Germany; launched: 2017).

#### 2.3.2. Image Collection and Analysis

*(1) Image Collection of Two-Dimensional Echocardiography (2DE)*. The following 2DE parameters were routinely measured and recorded with the S5-1 probe: interventricular septum diastolic dimension (IVSD), left ventricular end-diastolic posterior wall dimension (LVPWD), left ventricular end-diastolic volume (LVEDV), left ventricular end-systolic volume (LVESV), stroke volume (SV), and LVEF were measured. The left atrial volume index (LAVI) and left ventricular mass index (LVMI) were obtained by calculation. The early peak mitral orifice diastolic flow velocity (E) and left ventricular outflow tract pressure grade were measured using Doppler flow spectroscopy. The early peak mitral annular diastolic velocity (Ea), late peak mitral annular diastolic velocity (Aa), and isovolumic relaxation time (IVRT) were measured by tissue Doppler spectroscopy.

*(2) 3D-STE Analysis*. The X5-1 probe was used in a 2D echocardiographic mode to ensure a clear endocardial structure, and the 3D mode was used to display multiplanar reconstruction images. Four cardiac cycles were made to obtain two-chamber and four-chamber views of the left ventricle, and the images were stored. Image analysis was performed using 3D ultrasound image processing software, which divided the left ventricle into 16 segments and calculated the following global and segmental strain indicators: the global longitudinal strain (GLS), the global circumference strain (GCS), and the global radial strain (GRS), and the 16-segment regional longitudinal strain (RLS), regional circumferential strain, and regional radial strain.

### 2.4. Statistical Methods

SPSS 20.0 software was used for the statistical analysis. The measurement data were expressed as means ± standard deviations (*x* ± *s*). The independent samples *t*-test was used for comparisons between the two groups of measurement data. The countable data were expressed as frequencies and percentages, and the sensitivity and specificity of each relevant indicator were analyzed using a receiver operating characteristic (ROC) curve. Bland–Altman analysis was used for consistency testing. The test is divided into two parts. The first part is that the same observer carries out two GLS measurements as the internal measurement of the observer, and the second part is that two observers carry out one GLS measurement as the interobserver measurement. A *p* value<0.05 was considered statistically significant.

## 3. Results

### 3.1. Gene Detection

**Figure 2:**
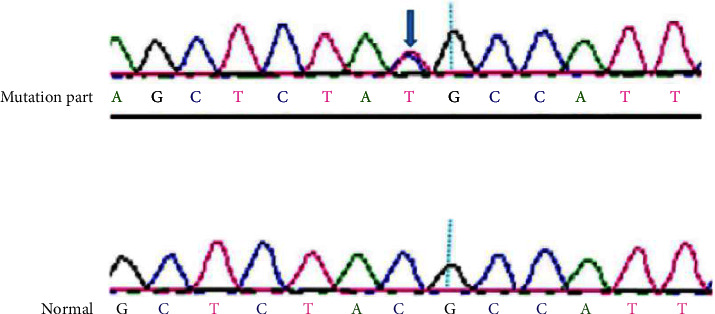
Peak atlas of the TTN gene mutation sequencing.

Six members of this pedigree carried the same TTN gene mutation locus, which was the TTN gene Val135643Ile heterozygous mutation, occurring in exon 360 of the human genome with a base substitution from C to T at position 106927. No abnormal mutations in this gene locus were found in the remaining 12 family members (see Figure 2).

### 3.2. A Comparison of the General Clinical Characteristics

**Figure 3:**
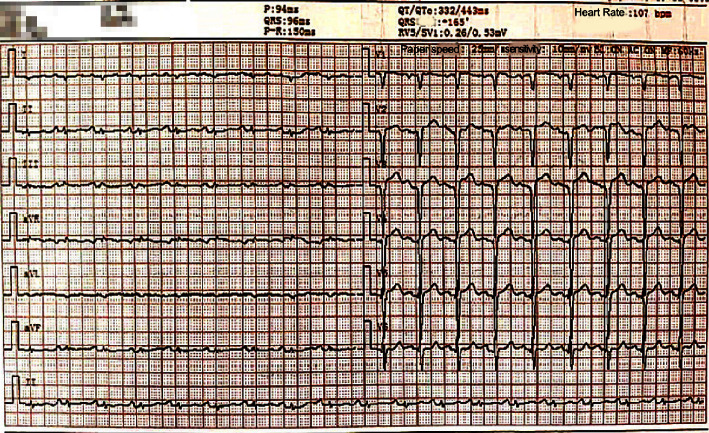
ECG photos of the proband.

**Figure 4:**
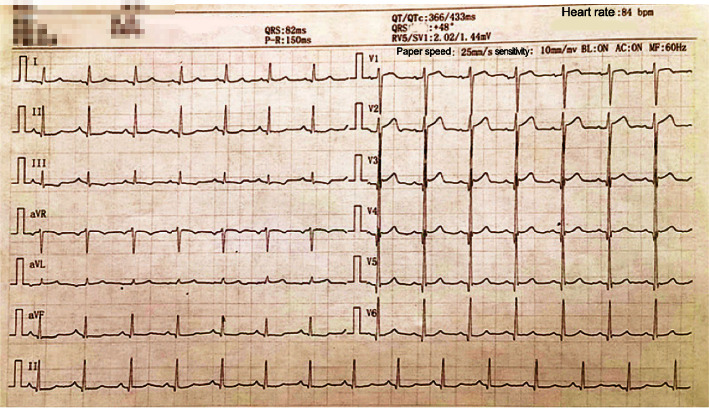
ECG of the older son of the proband.

① HCM: the proband (I-1) and the older son (II-1) carried the TTN gene mutations and were diagnosed with HCM using the echocardiography technique. The main clinical manifestations of the proband were abdominal distension and swelling of both lower limbs, with chest tightness, shortness of breath, weakness, and intermittent posterior back pain and discomfort. An electrocardiogram (ECG) showed a right deviation of the cardiac axis (+165°), low voltage in the limb leads, and a QS pattern in leads V1–V3 (see Figure 3). The older son mainly presented with shortness of breath after the activity with low T waves in leads I, II, and III and a VL on his ECG (see Figure 4). None of them were combined with clinical comorbidities. ② Asymptomatic pathogenic gene carriers: 4 members of the pedigree carried the TTN gene and had normal ECG results and no obvious cardiovascular clinical symptoms or clinical comorbidities. ③ Noncarriers: the remaining 12 members had negative gene detection results, normal ECG and echocardiogram results, and no cardiovascular clinical manifestations. One of them had high blood pressure.

There were no statistically significant differences between the two groups of family members in general clinical characteristics such as age, height, weight, body surface area, heart rate, and blood pressure (all *p* > 0.05) (see Table 1).

### 3.3. A Comparison of Conventional 2D Echocardiographic Indicators

In the pathogenic gene carrier group, I-1 showed a generalized thickening of the left ventricular wall, which was most obvious in the septum, of which the thickest part was approximately 19.6 mm in the basal segment of the anterior septum, without the obstruction of the left ventricular outflow. II-1 showed mainly a thickening of the basal segment of the ventricular septum of approximately 14.0 mm, with no thickening of the rest of the ventricular wall segments and no obstruction of the left ventricular outflow. Other members of the carrier group failed to meet the ventricular wall thickness for a diagnosis of HCM. Compared with the noncarrier group, the IVSD, LVPWD, and LVMI and E/Ea values were significantly higher while the Ea, Ea/Aa, and *E* values were significantly lower in the carrier group, and the differences were all statistically significant (all *p* < 0.05). However, the differences between the two groups in the remaining ultrasonic indicators were not significant (all *p* > 0.05) (see Table 2).

### 3.4. A Comparison of the Global 3D Strain Indicators

**Figure 5:**
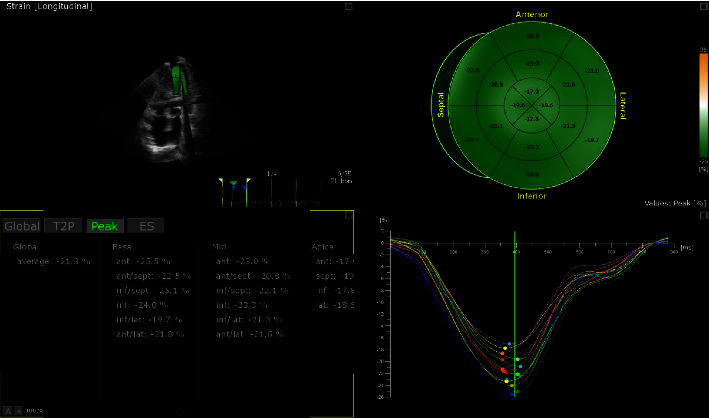
Gene carrier's illustration of the bull eye.

**Figure 6:**
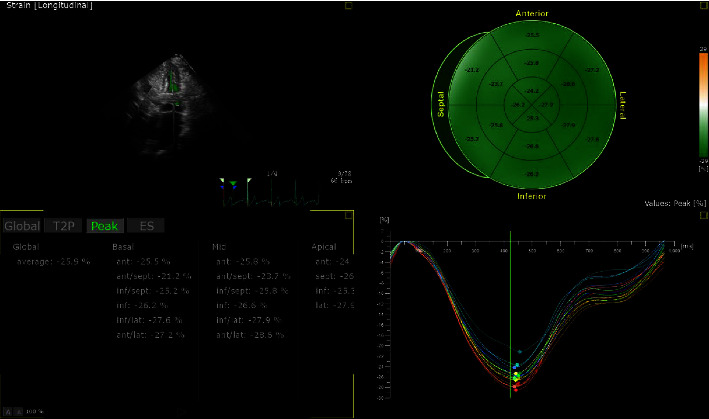
Noncarrier's illustration of the bull eye.

Compared with the noncarrier group, the GLS was significantly reduced in the carrier group (see Figures 5 and 6), and the difference was statistically significant (*p* < 0.05), while there was no significant difference between the groups in GRS and GCS (*p* > 0.05) (see Table 3).

### 3.5. A Comparison of the 16-Segment 3D Strain Indicators

In comparison with the noncarrier group, the carrier group showed a significantly reduced longitudinal strain in the basal segment of the anterior septum, the segments of the middle posterior septum, and the middle posterior wall (*p* < 0.05), while the remaining indicators in each segment were not significantly different (all *p* > 0.05) (see Table 4).

### 3.6. An ROC Curve Analysis of GLS and Conventional 2DE-Related Indicators

**Figure 7:**
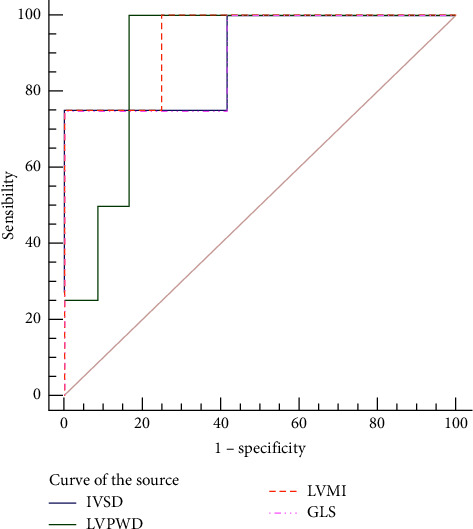
ROC curves of GLS and conventional 2D echocardiographic related indicators.

Among the indicators of the conventional 2DE and global 3D strain indicators, significant differences were found in IVSD, LVPWD, LVMI, and GLS values in the carrier group compared with the noncarrier group, and a ROC curve analysis of these parameters showed that GLS had the largest area under the curve (AUC), its sensitivity was better than LVPWD, and its specificity was better than IVSD and LVMI (see Table 5 and Figure 7).

### 3.7. Analysis of the Consistency Test

**Figure 8:**
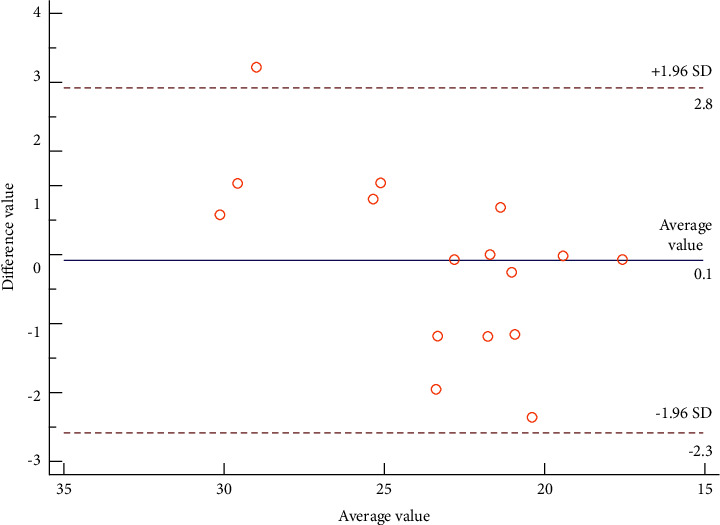
Analysis of the intraobserver consistency of GLS.

**Figure 9:**
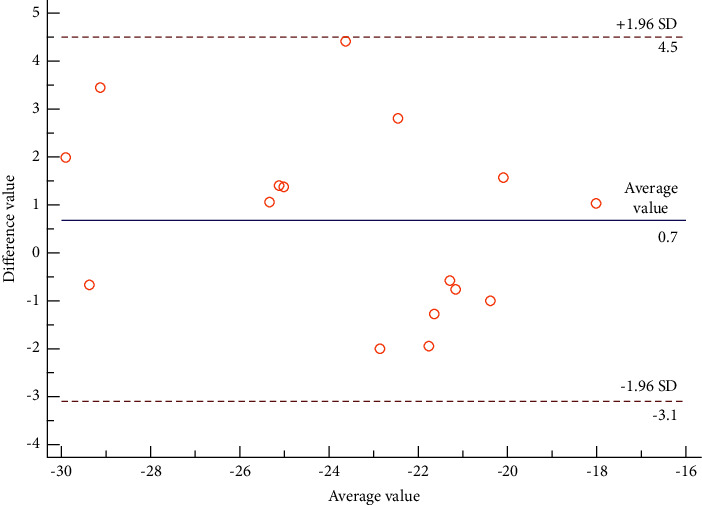
Analysis of the interobserver consistency of GLS.

The Bland–Altman consistency test was conducted on the GLS of the 16 subjects enrolled in the present study. This means that the results obtained from two measurements by the same observer and the results of measurements taken by two different observers were analyzed (see Figure 4). The average difference in the intraobserver GLS measurements was 0.14, with a 95% confidence interval (CI) of −2.55 to 2.84 and a repeatability coefficient of 12.62%. The average difference in the interobserver GLS measurements was 0.68, with a 95% CI of −3.10 to 4.46 and a coefficient of variation of 13.89%. The above results suggest that GLS has good consistency and repeatability within and between observers (see Figures 8 and 9).

## 4. Discussion

HCM is common in clinical practice, and gene detection and analysis techniques allow for the identification and characterization of pathogenic mutated genes in approximately 60% of patients with HCM. Among them, mutated genes such as MYBPC3, MYH7, TNNI3, TNNT2, TPM1, and PRKAG2 are common pathogenic genes in HCM. In recent years, with the use of high-throughput sequencing techniques, large samples of genes can be analyzed rapidly and a large number of relevant candidate genes can be screened, so that rare genetic mutations in HCM are constantly being identified and included in the list of pathogenic genes of HCM, thus expanding the diagnostic scope [7]. Gene detection has two main clinical applications. The first is for guiding the early diagnosis of patients with HCM and directing clinical intervention, and the second is for early screening and regular follow-up of asymptomatic pathogenic gene carriers in the pedigree.

In the present study, a total of 18 members of a pedigree of FHCM were enrolled as the subjects. Two members, including the proband, were diagnosed with HCM by conventional echocardiography, and four members were found, using gene detection, to have mutations in the TTN gene at the Val135643Ile locus, while the other 12 members of the family were not found to have mutations in this locus. Thus, it was confirmed that the FHCM in the present family was caused by the mutation at Val135643Ile in the TTN gene. The TTN gene has been shown to encode the largest protein in the human body [8]. The sarcomere is the basic structural unit that facilitates the contraction of the striated muscle, and an important component of the sarcomere is the TTN protein, which also belongs to the category encoding sarcomere contractile protein. Structurally, the TTN protein is a biological spring that spans half of the sarcomere and connects the Z-disc to the M-line [8]. It is a major determinant of passive tension and stretch in cardiac myocytes within the physiological length of the muscle and is also involved in the regulation of active contractility [9, 10]. Any modification of TTN at the transcriptional and translational levels of the gene may affect cardiac function by altering the passive and active tone of the sarcomere and cardiomyocyte. The large size of the TTN gene, the large number of repetitive sequences, and the broad nature of selective splicing have all made its detection and analysis difficult. Only a small number of the TTN mutations were found to be correlated with human myocardial disease in early studies [11]. Approximately 30% of patients with dilated cardiomyopathy were correlated with the TTN mutations, and only 1% of patients with HCM had mutations localized in TTN [12]. The results of the present study have confirmed that the rare TTN gene mutation locus Val135643Ile was a cause of FHCM in the present pedigree.

Compared with the noncarrier group, the IVSD, LVPWD, and LVMI values were significantly higher in the carrier group in the present study. This result indicated that in the present pedigree, although the LVPWD, IVSD, and LVMI values were within the normal range of the diagnostic criteria in the international guidelines, these indicators differed significantly from those of the pathogenic gene noncarriers. Thus, the carriers of the pathogenic gene showed relative hypertrophy of the ventricular wall due to abnormal expression of the pathogenic gene. Compared with the noncarrier group, the carrier group had significantly lower Ea, Ea/Aa, and *E* values and a significantly higher E/Ea value. This suggested that in asymptomatic pathogenic gene carriers with mutations at the Val135643Ile locus of the TTN gene, myocardial compliance was reduced and diastolic impairment had occurred in the left ventricle despite normal LVEF. This change was consistent with the changes in patients with HCM caused by other common pathogenic gene mutations and the pathophysiological changes in other cardiovascular diseases. In other words, the lower Ea, Aa, and *E* values with higher *E*/Ea values might reflect the reduced left ventricular diastolic function and changes in compliance [13].

At present, 3D-STE has been widely used in the early study of the left ventricular and left atrial dysfunction under related diseases and pathological conditions. For example, 3D-STE analysis shows that the overall longitudinal strain of the left ventricle in patients with euthyroid Hashimoto's thyroiditis (eHsT) is significantly depressed than that in the control group, so it is clear that eHsT has a negative effect on left ventricular myocardial systolic function [14]. Related studies also evaluated the three-dimensional strain parameters of left ventricular myocardium in patients with coronary artery disease (CAD) and found that CAD had a considerable adverse effect on the longitudinal strain of left ventricular myocardium [15]. In patients with aortic valve sclerosis (AVS), left ventricular global longitudinal strain (LV-GLS) and left ventricular global circumferential strain (LV-GCS) were significantly decreased [16].

In the present study, 3D-STE was used to analyze the global and regional systolic function of the left ventricle and the results showed that the global longitudinal strain of the left ventricle was significantly reduced in the carrier group compared with the noncarrier group, and the longitudinal strain in the basal segment of the anterior septum, the segments of the middle posterior septum, and the middle posterior wall was significantly reduced. This result suggested that asymptomatic TTN carriers had global and segmental abnormalities in the longitudinal myocardial systolic function of the left ventricle. The carrier group of the present pedigree had a marked thickening of the basal segment of the anterior septum, while the degree of myocardial hypertrophy was the main factor affecting myocardial contractile function, and the more pronounced the hypertrophy, the more severely the myocardium was damaged [17]. Due to the rearrangement and structural disorganization of the hypertrophic segment, the systolic function of the thickened segment in the ventricular wall was more significantly reduced. There were no statistically significant differences between the two groups in the global and segmental circumferential strain and longitudinal strain indicators, suggesting that although asymptomatic TTN pathogenic gene carriers showed a reduced longitudinal strain in the left ventricle as a whole and in some of the segments, both the global and segmental circumference and longitudinal strains remained relatively stable, which could be considered as a compensatory mechanism for the longitudinal strain injury to maintain the overall left ventricular systolic function within the normal range. The analysis of the results was based on the following structural characteristics and mechanical model of myocardial stratification: the subendocardial myocardial fibers follow a longitudinal path and gradually extend in a circumferential direction as they travel towards the middle layer of the ventricular wall, while the subepicardial fibers change to a reverse oblique path. The subendocardial myocardial fibers account for a relatively large proportion, approximately 70% of the overall myocardial fibers. Therefore, the longitudinal contraction of the left ventricular wall depends mainly on the subendocardial myocardial layer [18]. When hypoperfusion occurs in the subendocardial myocardium, the reduction in blood flow is more pronounced than in the subepicardial myocardium and is more likely to cause ischemia [19, 20], resulting in earlier impairment of the subendocardial myocardial systolic function. In addition, during left ventricular contraction, the subendocardial myocardium has greater wall thickening than the subepicardial myocardium, which requires more oxygen, and the subendocardial myocardium is less tolerant to ischemia [21]. The above characteristics make the left ventricular longitudinal strain a sensitive indicator for early detection of the abnormal subclinical systolic function.

The ROC curves showed that the 2D echocardiographic indicators, including IVSD, LVPWD, and LVMI values, and the 3D global strain indicators were useful for the early diagnosis of asymptomatic pathogenic gene carriers. In particular, the sensitivity of GLS was 75% and the specificity was 92%; its sensitivity was better than LVPWD, and its specificity was better than IVSD and LVMI. Therefore, it could be that a person is more likely to carry the TTN gene when the GLS is −21.24. Previous related studies [22, 23] found that the global 3D strain indicator GLS had a higher diagnostic value for asymptomatic pathogenic gene carriers with HCM than the related 2D ultrasonic indicators. Xu et al. [22] reported that the sensitivity of GLS in diagnosing asymptomatic pathogenic gene carriers was 71% and the specificity was 95%, which was close to the results of the present study. We know that asymptomatic carriers of pathogenic genes lack clinical symptoms and the performance of conventional 2DE is no different from that of normal people. Therefore, taking GLS as an important indicator for the early diagnosis of such carriers, its objectivity and repeatability are particularly important. To this end, we conducted a Bland–Altman conformance test. The results show that no matter the difference between the two measured values within the observer, the difference between the two measurements between the observers is within the consistency limit, indicating that GLS has good consistency and repeatability and can be applied in the early diagnosis and later follow-up for asymptomatic TTN gene carriers.

## 5. Conclusion

In this study, gene detection revealed the rare pathogenic gene TTN and the mutation locus of Val135643Ile to be the cause of HCM in this family, four of whom were asymptomatic pathogenic gene carriers. The study has shown that 3D-STE was able to sensitively identify early abnormalities of the global and regional left ventricular systolic function in the asymptomatic pathogenic gene carriers. Among several comparison indicators with significant abnormalities, GLS had the largest area under the curve (AUC); it is an indicator with good sensitivity and specificity, which suggests that subjects are most likely to carry the TTN gene when the GLS is −21.24. GLS measured by 3D-STE technology has good consistency and repeatability, which can provide an objective and accurate ultrasound diagnosis for early diagnosis and follow-up of such subjects in the future.

## 6. Limitations

This article already enrolled total 18 members from a family with familial HCM. The sample size was smaller from the statistical point of view, but it was not possible for this family to collect more subjects. We would like to continue to expand the sample size of more families with familial HCM in future related studies. Over time, with a larger sample size, it will be explored whether there are the same mutant genes with the same mutation sites or not the same mutation sites by high-throughput gene sequencing of gene detection technology.

## Figures and Tables

**Table 1:** Comparison of general clinical data between the two groups ($\bar{x}$ ± *s*).

Index	Noncarrier group (*n* = 12)	Carrier group (*n* = 4)	*p*
Age (years)	39.73 ± 12.81	40.96 ± 9.20	0.110
Height (cm)	161.69 ± 6.26	160.32 ± 10.55	0.416
Weight (Kg)	62.80 ± 7.52	63.82 ± 12.16	0.493
Body surface area (m^2^)	1.72 ± 0.46	1.79 ± 0.88	0.328
Heart rate (times/min)	72.73 ± 8.23	72.10 ± 7.94	0.486
Systolic pressure (mmHg)	110.14 ± 13.33	113.29 ± 10.89	0.560
Diastolic pressure (mmHg)	67.10 ± 2.12	68.10 ± 10.46	0.531

There was no significant difference between the two groups, *p* > 0.05.

**Table 2:** Comparison of two-dimensional echocardiography between the two groups ($\bar{x}$ ± *s*).

Index	Noncarrier group (*n* = 12)	Carrier group (*n* = 4)	*p*
IVSD (mm)	7.50 ± 0.70	**8.95** **±** **1.21**^*∗*^	**0.011**
LVPWD (mm)	7.40 ± 0.51	**8.91** **±** **0.93**^*∗*^	**0.042**
LVEDV (ml)	83.28 ± 15.96	81.52 ± 10.28	0.101
LVESV (ml)	28.36 ± 8.51	28.40 ± 6.25	0.280
LVEF (%)	65.64 ± 6.32	65.49 ± 5.25	0.196
SV (ml)	53.22 ± 9.86	52.77 ± 4.10	0.521
IVRT (ms)	76.51 ± 9.45	78.00 ± 20.14	0.793
LVMI (g/cm^2^)	46.04 ± 2.91	**52.03** **±** **5.22**^*∗*^	**0.017**
LAVI (ml/cm^2^)	18.10 ± 3.84	19.01 ± 9.84	0.602
Ea	9.41 ± 1.26	**6.87** **±** **0.76**^*∗*^	**0.002**
Ea/Aa	1.22 ± 0.34	**0.81** **±** **0.19**^*∗*^	**0.036**
E	70.61 ± 3.27	**56.42** **±** **6.21**^*∗*^	**0.000**
E/Ea	7.38 ± 0.52	**8.17** **±** **0.81**^*∗*^	**0.007**

Compared with the noncarrier group, ^*∗*^*p* < 0.05. The bold values are indexes, *p* < 0.05.

**Table 3:** Comparison of overall three-dimensional strain indexes between the two groups ($\bar{x}$ ± *s*).

Index	Noncarrier group (*n* = 12)	Carrier group (*n* = 4)	*p*
GLS (%)	−24.28 ± 2.99	−**20.11** **±** **2.23**^*∗*^	0.024
GCS (%)	−36.05 ± 3.98	−39.86 ± 7.76	0.211
GRS (%)	46.94 ± 5.61	53.02 ± 9.92	0.307

Compared with the noncarrier group, ^*∗*^*p* < 0.05. GLS: global longitudinal strain, GCS: global circumference strain, and GRS: global radial strain.

**Table 4:** Comparison of three-dimensional strain indexes of 16 segments between the two groups ($\bar{x}$ ± *s*).

Groups	RLS (%)		RCS (%)		RRS (%)	
	Noncarrier group (*n* = 12)	Carrier group (*n* = 4)	Noncarrier group (*n* = 12)	Carrier group (*n* = 4)	Noncarrier group (*n* = 12)	Carrier group (*n* = 4)
*Apex segment*						
Antetheca	−15.62 ± 3.13	−10.35 ± 2.16	−21.46 ± 2.28	−23.96 ± 5.17	56.12 ± 10.58	59.92 ± 18.44
Side wall	−17.99 ± 0.97	−16.10 ± 1.16	−20.51 ± 3.71	−15.62 ± 6.41	63.21 ± 9.12	45.37 ± 17.12
Inferior wall	−20.23 ± 5.14	−18.65 ± 1.44	−25.94 ± 3.65	−30.55 ± 4.39	102.99 ± 29.06	99.14 ± 20.13
Interventricular septum	−19.67 ± 3.76	−13.67 ± 1.43	−12.21 ± 5.35	−15.57 ± 6.27	93.25 ± 28.10	88.85 ± 20.96

*Mid-segment*						
Anteroseptum	−18.72 ± 0.59	−18.14 ± 2.96	−16.52 ± 3.93	−15.97 ± 5.49	58.15 ± 13.21	45.91 ± 12.05
Antetheca	−21.71 ± 2.16	−20.33 ± 1.88	−24.82 ± 3.14	−25.54 ± 6.65	80.53 ± 9.22	74.16 ± 2.43
Side wall	−20.63 ± 1.75	−18.87 ± 1.21	−23.39 ± 2.10	−21.48 ± 2.66	75.59 ± 7.16	69.88 ± 13.10
Posterior wall	−18.04 ± 0.81	**−9.71** **±** **1.32**^*∗*^	−27.36 ± 1.84	−26.17 ± 7.32	82.95 ± 10.42	55.61 ± 9.33
Inferior wall	−21.58 ± 1.03	−17.61 ± 1.12	−28.93 ± 4.62	−25−15 ± 3.61	96.18 ± 15.24	81.16 ± 14.77
Posterior-septal	−18.54 ± 2.69	**−13.09** **±** **0.28**^*∗*^	−20.31 ± 4.62	−21.75 ± 4.49	80.26 ± 11.65	79.22 ± 10.37

*Basal segment*						
Anteroseptum	−25.67 ± 4.14	**−12.62** **±** **3.12**^*∗*^	−21.48 ± 4.03	−19.96 ± 3.15	92.13 ± 18.43	79.91 ± 11.42
Antetheca	−26.48 ± 1.71	−22.23 ± 3.16	−22.34 ± 3.52	−20.77 ± 3.40	103.87 ± 31.80	90.55 ± 10.74
Side wall	−21.01 ± 2.11	−19.06 ± 2.13	−20.89 ± 2.06	−19.02 ± 4.81	82.92 ± 8.11	79.49 ± 10.69
Posterior wall	−20.55 ± 1.26	−21.51 ± 2.92	−20.49 ± 2.18	−21.63 ± 0.87	65.25 ± 20.16	70.51 ± 10.77
Inferior wall	−22.62 ± 4.84	−23.31 ± 1.83	−17.91 ± 3.13	−19.20 ± 3.55	96.01 ± 22.00	89.14 ± 12.59
Posterior-septal	−20.58 ± 3.13	−18.58 ± 2.04	−16.37 ± 4.03	−15.92 ± 5.86	70.49 ± 10.40	68.14 ± 20.99

Compared with the noncarrier group, ^*∗*^*p* < 0.05. RLS: regional longitudinal strain, RCS: regional circumference strain, and RRS: regional radial strain. The bold values are indexes, *p* < 0.05.

**Table 5:** ROC curve analysis results of GLS and conventional 2DE-related indicators.

Index	Cut-off value	Area under the curve	95%CI	*p*	Sensitivity (%)	Specificity (%)
IVSD	8.22	0.85	0.64–0.99	0.021	75	83
LVPWD	8.15	0.83	0.69–0.99	0.011	69	92
LVMI	50.50	0.89	0.64–0.99	0.021	75	90
GLS	−21.24	0.89	0.64–0.99	0.021	75	92

IVSD: interventricular septum diastolic dimension, LVPWD: left ventricular end-diastolic posterior wall dimension, LVMI: left ventricular mass index, and GLS: global longitudinal strain.

## Data Availability

The data sets used and/or analyzed during the current study available from the corresponding author on reasonable request.
